# PD-1 pathway inhibitors: The next generation of immunotherapy for advanced melanoma

**DOI:** 10.18632/oncotarget.2980

**Published:** 2015-02-27

**Authors:** Jason J. Luke, Patrick A. Ott

**Affiliations:** ^1^ Section of Hematology/Oncology, University of Chicago, Chicago, IL, USA; ^2^ Melanoma Disease Center, Dana Farber Cancer Institute and Harvard Medical School, Boston, MA, USA

**Keywords:** immunotherapy, melanoma, programmed death 1 pathway, PD-1, adverse events

## Abstract

Checkpoint inhibitors are revolutionizing treatment options and expectations for patients with melanoma. Ipilimumab, a monoclonal antibody against cytotoxic T-lymphocyte-associated antigen 4 (CTLA-4), was the first approved checkpoint inhibitor. Emerging long-term data indicate that approximately 20% of ipilimumab-treated patients achieve long-term survival. The first programmed death 1 (PD-1) inhibitor, pembrolizumab, was recently approved by the United States Food and Drug Administration for the treatment of melanoma; nivolumab was previously approved in Japan. PD-1 inhibitors are also poised to become standard of care treatment for other cancers, including non-small cell lung cancer, renal cell carcinoma and Hodgkin's lymphoma. Immunotherapy using checkpoint inhibition is a different treatment approach to chemotherapy and targeted agents: instead of directly acting on the tumor to induce tumor cell death, checkpoint inhibitors enhance or *de novo* stimulate antitumor immune responses to eliminate cancer cells. Initial data suggest that objective anti-tumor response rates may be higher with anti-PD-1 agents compared with ipilimumab and the safety profile may be more tolerable. This review explores the development and next steps for PD-1 pathway inhibitors, including discussion of their novel mechanism of action and clinical data to-date, with a focus on melanoma.

## INTRODUCTION

Despite recent clinical advancements, the treatment of advanced melanoma continues to represent a significant challenge. Historically, the 5-year survival rate for patients with stage IV disease was approximately 6% [1–3]. However, agents introduced in recent years have substantially improved the outlook for patients with melanoma. For example, BRAF or MEK inhibitors such as vemurafenib, dabrafenib or trametinib, which are indicated in the approximately 50–60% of patients with melanoma harboring a *BRAF^V600E/K^* mutation, are associated with high response rates (~20–80%), prolonged progression-free survival (PFS) (5–9 months), and improved overall survival (OS) [4–7]. Unfortunately, most if not all patients receiving BRAF or MEK inhibitors eventually develop resistant tumors leading to disease progression [4, 6, 8–11].

In contrast to these kinase inhibitors, a second major advance in clinical therapeutics came with the development of ipilimumab and tremelimumab; monoclonal antibodies that can induce an antitumor immune response by blocking the checkpoint molecule cytotoxic T-lymphocyte-associated antigen 4 (CTLA-4) [12, 13]. Although these anti-CTLA-4 antibodies have modest response rates in the range of 10% [12, 13], ipilimumab significantly improves OS, with a subset of patients experiencing long-term survival benefit [14]. In a phase III trial, tremelimumab was not associated with an improvement in OS [13], and tremelimumab is not currently approved for the treatment of melanoma. Across clinical trials, survival for ipilimumab-treated patients begins to separate from those patients treated in control arms at around 4–6 months, and improved survival rates are seen at 1, 2, and 3 years [12, 14, 15](Table 1 [4, 7, 10, 12, 13, 16–25]). Further, in aggregating data for patients treated with ipilimumab, it appears that there may be a plateau in survival at approximately 3 years. Thereafter, patients who remain alive at 3 years may experience a persistent long-term survival benefit, including some patients who have been followed for up to 10 years [14, 26]. While BRAF inhibitors also provide improved OS over chemotherapy, similar long-term follow up is not yet available with these agents. It is possible that the long-term effect seen with ipilimumab is unique to immunotherapeutic approaches, as similar long-term survival in a subset of patients has been previously reported with interleukin (IL)-2 therapy [27]. These observations suggest that in some patients treated with immunotherapy, cancer can be kept in check for an extended period of time, which may be a consequence of an effective and ongoing immune response. The next generation of checkpoint inhibitors, used either as single agents or in combination regimens, offers the promise of extending clinical benefits to a larger number of patients.

**Table 1:** Mechanism of action of anticancer agents in melanoma and association with response patterns and safety profile [4, 7, 10, 12, 13, 16–25]

Type	Examples	Mechanism of Action	Antitumor Activity	Toxicities	Reference(s)
Chemotherapy	Dacarbazine	Induces DNA damage and death of dividing cells	Directly cytotoxic effects cause tumor regression or non-progression	Off-target effects responsible for neutropenia, thrombocytopenia, and leukopenia	[7,16]
Targeted agents (e.g. BRAF and MEK kinase inhibitors)	Vemurafenib, dabrafenib, trametinib	Inhibit mutated signaling pathway (BRAF/MAPK) driving melanoma proliferation	Directly antiproliferative effects lead to tumor regression or non-progression	Effects on wild type BRAF and CRAF likely responsible for skin toxicities	[4,7,10]
CTLA-4 checkpoint inhibitors	Ipilimumab, tremelimumab^a^	Inhibit CTLA-4-mediated T-cell inhibition; increases T-cell proliferation; depletion/inhibition of regulatory T cells	Reactivated antitumor immune response can lead to immediate or delayed tumor regression or non-progression	T-cell activation and proliferation can lead to immunologic AEs	[12, 13, 17–19]
PD-1 checkpoint inhibitors	Pembrolizumab, nivolumab^a^, pidilizumab^a^	Inhibit PD-1:PD-L1– and PD-1:PD-L2– mediated T-cell inhibition	Restored antitumor immune response can lead to immediate or delayed tumor regression or non-progression	T-cell activation can lead to immunologic AEs	[18, 20–22]
PD-L1 checkpoint inhibitors	MPDL3280A^a^, MEDI4736^a^, MSB0010718C ^a^	Inhibit PD-1:PD-L1–mediated T-cell inhibition	Restored antitumor immune response can lead to immediate or delayed tumor regression or non-progression	T-cell activation can lead to immunologic AEs	[18, 23–25]

a Not FDA-approved for the treatment of patients with melanoma at the time of writing.

### Mechanisms of action of immune checkpoint inhibitors

The goal of immunotherapy is to elicit or enhance antitumor immune responses. Whereas BRAF and MEK targeted agents specifically inhibit a node in the MAPK signaling pathway that can eventually be overcome by tumor mutation, cancer immunotherapy has the potential to induce the inherent capacity of the immune system to adapt to mutational tumor changes. Though cancer immunotherapy approaches have been pursued for decades and have been successful in some cases (e.g. IL-2 in melanoma), checkpoint inhibition and, in particular, PD-1/PD-L1 blockade, is the first strategy that is poised to impact the outcome in cancer patients on a broader scale.

**Figure 1:**
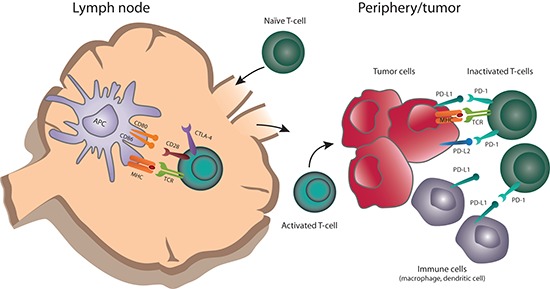
Role of CTLA-4 and PD-1 in antitumor immune responses Naïve T cells are primed by antigens presented by APCs in the context of MHC (signal 1), as well as co-stimulatory binding of CD28 to B7 (CD80/86) (signal 2). T cells upregulate CTLA-4 shortly after activation. Ligation of CTLA-4 with CD80 or CD86 limits T-cell activation and proliferation. Activated T cells traffic to the periphery and encounter tumor antigens at the tumor site. PD-1 is upregulated on T cells after prolonged activation; binding to PD-1 ligands (PD-L1 or PD-L2) expressed by tumor or other immune cells, including macrophages and dendritic cells, causes T-cell activation and dampens an ongoing antitumor immune response.

Under physiologic conditions, both stimulatory and inhibitory pathways regulate the inflammatory immune response to pathogens and maintain tolerance to self-antigens. These are regulated by a diverse set of immune checkpoints, thereby protecting healthy tissues from damage [18]. These checkpoints can be co-opted by malignant tumors to dampen the immune response and evade destruction by the immune system [18]. The CTLA-4 and PD-1pathways have been the initial focus of anticancer agent development (Figure 1); agents targeting other pathways are also in development [18].

Generally, the CTLA-4 and PD-1 pathways operate at different stages of the immune response (Figure 1) [28]. CTLA-4 modulates the immune response early—at the time of T-cell activation by antigen presenting cells (APCs). T cells are activated by antigen presented on APC in the context of major histocompatibility complex (MHC) (signal 1), as well as co-stimulatory binding of CD28 to B7 (CD80/86) (signal 2). Upon T-cell activation, CTLA-4 is trafficked from the Golgi apparatus to the plasma membrane where it out-competes CD28 in binding to B7 ligands on APCs due to its much higher binding affinity. CTLA-4 binding to B7 ligands inhibits further T-cell activation, limiting the time for T-cell activity. In this way, the magnitude and duration of initial immune responses is physiologically controlled.

**Figure 2:**
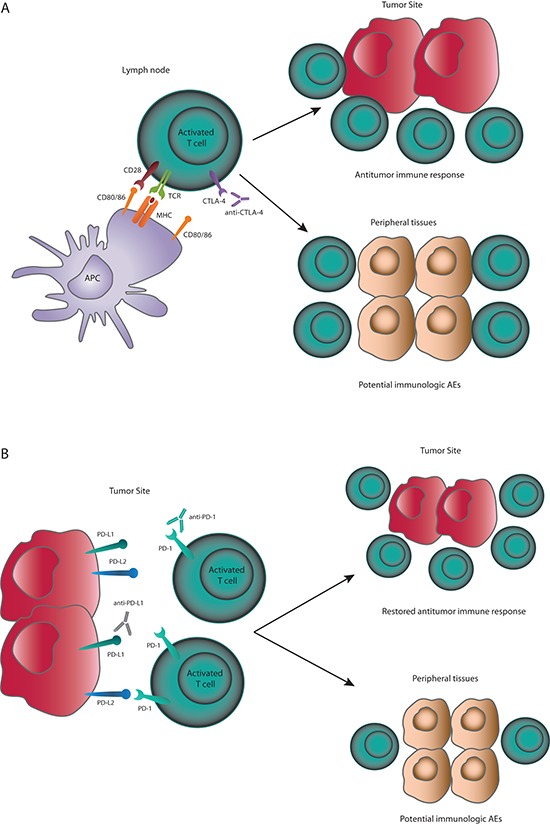
(A) CTLA-4 checkpoint inhibition CTLA-4 inhibition prevents early deactivation of T cells responding to tumor antigens presented by APCs. Activated T cells can migrate to the tumor site and mount effective antitumor immune responses. Activation of T cells with cross-reactivity to host antigens may cause immunologic AEs. **(B)** PD-1 and PD-L1 checkpoint inhibition. PD-1 checkpoint inhibitors will prevent PD-1:PD-L1– and PD-1:PD-L2–mediated deactivation of T cells. PD-L1 checkpoint inhibitors will prevent PD-1:PD-L1– mediated deactivation of T cells. PD-1 pathway inhibition can restore antitumor immune responses directly at the tumor site and also facilitate T-cell activation in lymph nodes or other sites. Activation of T cells with cross-reactivity to host antigens may cause immunologic AEs.

In the setting of cancer, inhibiting CTLA-4 may lead to continued activation of a larger number of T cells, resulting in more effective antitumor responses (Figure 2A). Preclinical evidence suggests that anti-CTLA-4 antibody can also deplete or inhibit regulatory T cells present in the tumor [19, 29]. CTLA-4 blockade has the potential to activate T cells that are specific for a wide range of antigens, including self-antigens, or deplete regulatory T cells that control autoreactive effector T cells, which may explain the autoimmune-like toxicities observed with CTLA-4 blockade.

In contrast to the effect of CTLA-4 on early T-cell activation, the PD-1 pathway appears to impact the T-cell response at the (later) effector stage (Figure 1). PD-1 is upregulated on T cells after persistent antigen exposure, typically in response to chronic infections or tumors. PD-L1 and PD-L2, the ligands for PD-1, can be expressed by tumor cells, as well as several other hematopoietic and non-hematopoietic cell types. Expression of PD-L1 and PD-L2 is induced by inflammatory cytokines, predominately interferon-γ [30]. In tumors, oncogenic signaling pathways can also upregulate PD-L1 expression [31]. When PD-1 binds its ligand, the T cell receives an inhibitory signal. Over time, chronic inhibition via PD-1:PD-L1 in tumor leads to T-cell anergy and blockade of a productive antitumor immune response [32].

Expression of PD-1 ligands is a mechanism for tumors to evade antitumor immune responses [30]. In many tumor types, tumor infiltrating lymphocytes (TILs) have been observed in both primary and metastatic specimens, indicating immune recognition of cancer cells. The presence of these TILs has been associated with improved outcomes in several cancers, including melanoma and renal cell carcinoma [33, 34]. Blockade of the PD-1/PD-L1 axis may restore the activity of TILs that have become quiescent as a result of PD-L1 ligation (Figure 2B).

Whereas CTLA-4 is widely expressed by T cells, PD-1 is expressed by activated T cells that have developed an “exhausted” or near-anergic state. PD-1 pathway blockade, in contrast to CTLA-4 blockade, may thus enhance activation of T cells with greater selectivity for tumors. However, human tumors can also express PD-L2, and this ligand has been found to be a negative prognostic factor in some cancers [30, 35]. PD-1 has a higher affinity for PD-L2 than for PD-L1, although whether this leads to differences in signaling or T-cell functions is not known [35]. PD-L2 is expressed in a number of tissues, most notably the lung, where it dampens the immune response to self-antigens and prevents autoimmunity [36]. It has been postulated that for selective blockade of PD-1:PD-L1 binding, keeping PD-1:PD-L2 interactions intact could offer a benefit in terms of reduced toxicities [37]. However, to date, a differential toxicity profile has not been observed between anti-PD-1 and anti-PD-L1 antibodies, and no direct comparisons from randomized trials are available.

Monoclonal antibodies directed at PD-1 (nivolumab, pembrolizumab, pidilizumab [CT-011]) are designed to prevent PD-1 from binding both PD-L1 and PD-L2 (Table 1 [4, 7, 10, 12, 13, 16–25]). PD-L1 inhibitors (MEDI4736, MPDL3280A, and MSB0010718C) block PD-1:PD-L1 binding, but not PD-1:PD-L2 binding. PD-L1 blockade also inhibits the binding of PD-L1 to CD80, which is expressed on activated T cells. The implication of this additional effect is not clear, as the role of PD-L1:CD80 interaction is not fully understood [18]. Pembrolizumab was approved by the United States Food and Drug Administration (FDA) in September 2014 for the treatment of patients with unresectable or metastatic melanoma with disease progression following ipilimumab and, if *BRAF^V600^* mutant, a BRAF inhibitor. Nivolumab was approved in Japan for the treatment of patients with unresectable melanoma in July 2014. The other PD-1 and PD-L1 directed agents are currently in Phase I–III clinical trials in multiple tumor types.

### Clinical response patterns

Rapid and dramatic responses have been observed with oncogene-directed treatments, such as BRAF inhibitors in melanoma and epidermal growth factor receptor (EGFR) inhibitors in non-small cell lung cancer. These agents specifically inhibit oncogenic signaling pathways. However, response duration with these agents has been modest. For example, the median PFS of BRAF inhibitors in *BRAF^V600E/K^* mutant melanoma ranges from 5–8 months [4, 8, 10, 11]. With dual BRAF and MEK blockade, PFS is longer, approximately 9 months [6, 9].

Checkpoint inhibitors display a range of response patterns, which may reflect the complexities of inducing a tumor-directed immune response and the individuality of a patient's immune system and tumor. Response kinetics may also depend on which pathway is inhibited. In theory, a patient with extensive tumor infiltration of PD-1-expressing T cells could have a rapid response with a PD-1 pathway inhibitor. In contrast, a patient with low numbers of pre-existing tumor-specific T cells could have a delayed or no response to PD-1 or CTLA-4 pathway blockade. Late or delayed responses occurring months to years after treatment initiation have been described with checkpoint inhibitors [17, 38–40]. In the case of a delayed response, tumor size may initially increase—as a result of either true tumor growth or increased tumor volume due to infiltration by immune cells—prior to subsequent tumor regression. As such, PFS based on traditionally used response criteria, such as RECIST, may not be the most appropriate efficacy measure with immunotherapies. To guide clinical practice, expert opinion has suggested the use of modified response criteria for immunotherapies, termed immune-related response criteria (irRC). These response criteria take into account the potential for increase in tumor size or number of lesions prior to declaration of progressive disease (Table 2 [12, 17, 21, 38, 40–46]). It has been suggested that RECIST may underestimate the benefit of PD-1 inhibitors in approximately 10% of patients relative to irRC [41].

**Table 2:** Select immunologic adverse events reported in patients with melanoma receiving checkpoint inhibitors [12, 17, 21, 38, 40–46]

Type	Examples	Frequency (All Grades)	Frequency (Grade ≥ 3)	Typical Timing of First Occurrence^a^	Treatment Approaches for Grade ≥ 3 AEs^b^	Typical Time to Resolution (Grades ≥ 2)
Dermatologic	Rash, pruritus, vitiligo	21–50%	≤ 4%	2–3 weeks	Dermatologist evaluation; drug interruption or discontinuation and systemic corticosteroids	≤ 3 months
Gastrointestinal	Diarrhea, colitis	Ipilimumab: 30–35% PD-1 inhibitors: 17–20%	Ipilimumab: 5–8% PD-1 inhibitors: ≤ 2%	5–6 weeks	Gastrointestinal consultation; colonoscopy may be considered; evaluation to rule out infection; drug discontinuation and systemic corticosteroids with≥30 day taper; infliximab^c^	≤ 3 months
Endocrine	Hypothyroidism, hyperthyroidism, hypophysitis, adrenal insufficiency	4–13%	≤ 1%	8–9 weeks	Endocrinologist evaluation; drug interruption or discontinuation; systemic corticosteroids and/or hormone replacement therapy	> 4 months; may be irreversible
Hepatic	Elevated ALT, elevated AST	2–9%	≤ 2%	6–8 weeks	Drug discontinuation and systemic corticosteroids; mycophenolate mofetil or other immunosuppressants per local guidelines	≤ 3 months
Pulmonary	Pneumonitis	≤ 4%	≤ 2%	6–12 weeks^d^	Drug interruption or discontinuation and systemic corticosteroids; additional immunosuppressant therapy as needed^e^	> 4 months in one patient^d^
Ocular	Conjunctivitis, scleritis, uveitis, Graves' ophthalmopathy	≤ 1%	< 1%	^d^	Drug interruption or discontinuation and topical or systemic corticosteroids; ophthalmologist consultation as needed	≤ 3 months^d^
Neurologic	Myopathy, Guillain-Barré syndrome, myasthenia gravis	< 1%	< 1%	d	Neurologist evaluation; drug discontinuation and systemic corticosteroids; IVIG or other immunosuppressants per local guidelines	d

a After treatment initiation. Individual patient experiences will vary.

b With the exception of endocrinopathies, add prophylactic antibiotics for opportunistic infections. Patients on IV steroids may be switched to an equivalent dose of oral corticosteroids (e.g. prednisone) at start of tapering or earlier, once sustained clinical improvement is observed. The lower bioavailability of oral corticosteroids should be taken into account.

c Unless contraindicated; should not be used in cases of perforation or sepsis.

d Information is limited due to small numbers of cases.

e Infliximab, cyclophosphamide, IVIG, or mycophenolate mofetil.

Abbreviations: ALT, alanine aminotransferase; AST, aspartate aminotransferase.

In clinical trials of ipilimumab, responses predominately occurred by 12 weeks, though there was a subset of patients with delayed response [47]. Further, some patients had an initial partial response that later converted to a complete response; the average time to reach a complete response was 30 months [47]. At the FDA-approved dose of pembrolizumab (2 mg/kg every 3 weeks), an open-label phase I trial reported an ORR of 26% in patients with ipilimumab-refractory advanced melanoma. The majority of patients had a response by the time of first assessment at 3 months (12 weeks); the median time to response was 3 months (range: 2.8 to 9) [48]. In a phase III trial of nivolumab (3 mg/kg, every 2 weeks) in patients with untreated metastatic melanoma, the ORR was 40% (*versus* 14% with dacarbazine). The median time to response with nivolumab was 2.1 months (range: 1.2 to 7.6), similar to that of dacarbazine (median 2.1 months; range: 1.8 to 3.6) [46].

In addition to delayed responses, treatment with checkpoint inhibition has been associated with durable, long-lasting responses, even after discontinuing therapy in some patients. In the phase I trial of nivolumab, the vmedian duration of responses was 99 weeks (range, 17+ to 117+ weeks), and 56% (19/34) of responses were ongoing at the time of last analysis (range of follow-up of responding patients, approximately 32–144 weeks, median follow up not disclosed) [20]. With shorter follow-up in the pembrolizumab trial, the median response duration had not been reached (range, 6+ to 76+ weeks), and 88% of responses were ongoing (range of follow-up of responding patients, approximately 18–88 weeks, median follow up not disclosed) [40]. Many patients who have discontinued immunotherapy (either anti-PD-1 or anti-CTLA-4) for reasons other than disease progression had persistent responses, indicative of a sustained antitumor immune response. In the phase 1 trial of nivolumab, 52% (11/21) of responding patients who discontinued therapy early had responses lasting ≥ 24 weeks off therapy [20]. Of these 11 patients, 7 (64%) had ongoing responses at the time of analysis.

### Survival with checkpoint inhibitors

In clinical practice, ipilimumab is administered at 3 mg/kg every 3 weeks for a total of 4 doses, based on the approved FDA label [49]. There is speculation in the field as to whether a higher dose (10 mg/kg) or the possibility of maintenance administration (every 12 weeks after the initial 4 doses) could influence the activity of the drug, and clinical trials are ongoing to investigate these possibilities. Nevertheless, the approved regimen has led to > 5-year survival in some heavily pretreated patients [14, 26, 47]. The durability and adaptability of tumor responses among patients who discontinued nivolumab and pembrolizumab therapy may be similar to that observed with ipilimumab, and may also explain partial or complete responses observed in patients who received PD-1 inhibitors after ipilimumab [40, 50].

Survival data with PD-1 inhibitors are promising. Significantly improved survival was seen with nivolumab *versus* dacarbazine in patients with previously untreated metastatic melanoma in a randomized phase III trial [46]. The 1-year survival rate was 73% with nivolumab and 42% with dacarbazine, and median PFS was 5.1 months and 2.2 months, respectively. In the phase I trial of pembrolizumab, median OS was not reached at the time of analysis, and 1-year and 18-month survival rates were 69% and 62%, respectively. In this study, 48% of patients had received ≥ 2 prior therapies [40]. Median OS was 17.3 months for all patients treated in the nivolumab phase I trial, 62% of whom had received ≥ 2 prior therapies, and 1-, 2- and 3-year survival rates were 63%, 48%, and 41%, respectively [20].

### Safety profile with checkpoint inhibitors

#### Safety with monotherapy

Related to the immune mechanism of antitumor activity, checkpoint inhibitors are associated with immune-related adverse events (irAEs). While different from those of targeted agents and chemotherapy, the safety profiles of checkpoint inhibitors are typically manageable and tolerable for most patients. Since these therapies induce tumor regression by stimulation of immune responses, side effects may be caused by activating potentially self-reactive T cells (Figure 2A and 2B). One exception to this is hypophysitis, which is reported in about 4% of patients receiving ipilimumab, and is attributed to ectopic expression of CTLA-4 in the pituitary gland, leading to ipilimumab binding to endocrine cells, complement fixation, and inflammation [51].

In the phase III study of ipilimumab monotherapy compared with the gp100 vaccine or ipiliumumab plus gp100 vaccine, the incidence of grade 3–4 adverse events (AEs) in the ipilimumab monotherapy arm was 24% [12]. In comparison, the incidence of treatment-related grade 3–4 AEs in the phase I nivolumab and pembrolizumab trials were 22% and 12%, respectively [21, 40]. In the phase III nivolumab trial, reported rates of treatment-related grade 3–4 AEs were 12% with nivolumab and 18% with dacarbazine [46]. The most commonly-reported treatment-related AE with ipilimumab is fatigue, while the most common clinically significant immunologic AEs include diarrhea/colitis, rash/pruritus, endocrinopathies, and hepatitis (Table 2 [12, 17, 21, 35, 40–46]). While fatigue is also common with PD-1 inhibitors, diarrhea may be less common as compared with ipilimumab (Table 2 [12, 17, 21, 38, 40–46]). In contrast, pneumonitis, though infrequent, may be more common with PD-1 pathway inhibitors than with ipilimumab. The rate of grade 3–4 pneumonitis was 2% or less in trials of PD-1 pathway inhibitors [22, 52, 53]. However, pneumonitis was the cause of death in 3 patients with cancer who received nivolumab; no deaths occurred in patients with melanoma [21].

**Figure 3:**
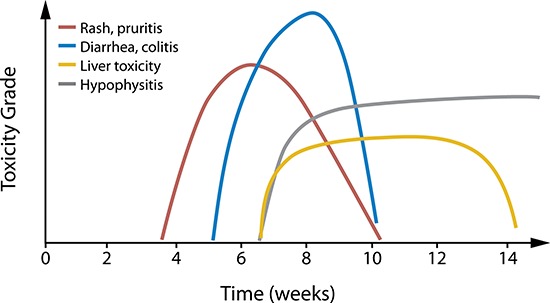
Kinetics of irAEs in ipilimumab-treated patients The overall approximate timing and relative grade of the most common irAEs in ipilimumab-treated melanoma patients is depicted. Individual patient experiences vary. Reprinted with permission. © 2012 American Society of Clinical Oncology. All rights reserved. Weber JS et al: *J Clin Oncol*. 30 (21), 2012: 2691–2697 [38].

Most irAEs with checkpoint inhibitors occur during the first 2 to 6 months of treatment, but can occur anytime, even after treatment discontinuation [21, 42, 45] (Table 2 [12, 17, 21, 38, 40–46]). The general timing and grade of irAEs with ipilimumab have been described by Weber and colleagues (Figure 3 [38]). However, whether PD-1 pathway inhibitors have similar AE kinetics is not currently clear. With chemotherapies, toxicities can be cumulative, but this may not be the case with immunotherapies. In patients who received up to 2 years of nivolumab treatment, there was no evidence of increasing toxicity with increased drug exposure [21]. Furthermore, no maximum tolerated dose was reached in the phase I studies of nivolumab or pembrolizumab [54, 55].

#### Safety with checkpoint blockade combination therapy

Preclinical animal studies suggest that dual checkpoint blockade with anti-CTLA-4, anti-PD-1, and anti-PD-L1 antibodies leads to stronger immune stimulation and enhanced antitumor activity [56]. In a phase I trial, concurrent checkpoint blockade with both anti-CTLA-4 (ipilimumab) and anti-PD-1 (nivolumab) antibodies showed increased efficacy over what has been observed with single agent therapy. The ORR with combination therapy was 42% across all tested doses, *versus* 11% and 32% reported in trials of ipilimumab and nivolumab monotherapy, respectively [12, 20, 57], and 42% of patients had ≥ 80% tumor reduction at 36 weeks [57]. Preliminary survival rates at 1 and 2 years were 85% and 79%, respectively, for the combination regimen, which compared favorably with the reported 1- and 2-year rates of 46% and 24% for ipilimumab and 63% and 48% for nivolumab [12, 20, 57].

Combining CTLA-4 and PD-1 pathway blockade resulted in a higher incidence of AEs compared with single agent therapy, whereby the types of AEs were similar to what has been observed with ipilimumab or nivolumab alone. Sixty-two percent of patients with concurrent ipilimumab plus nivolumab therapy experienced a grade 3–4 treatment-related AE, and 23% of patients discontinued therapy due to a treatment-related AE [57]. The majority of grade 3–4 treatment-related AEs were laboratory abnormalities, including elevated aspartate aminotransferase (13%), lipase (13%), alanine aminotransferase (11%), creatinine (6%), and diarrhea (6%) [50]. Multiple ongoing studies are evaluating different checkpoint combinations and doses to optimize the risk/benefit profile of dual checkpoint blockade [23].

Since pembrolizumab is approved for patients with progression after ipilimumab therapy, and other PD-1 targeting agents are likely to be approved soon, patients who have previously received ipilimumab may subsequently receive a different checkpoint inhibitor. To date, trials of PD-1 pathway inhibitors in patients who previously received ipilimumab have not shown an increased risk of select irAEs [40, 44, 48, 58]. In one study, patients who had previously experienced grade 3 irAEs with ipilimumab did not experience similar AEs when treated with nivolumab [44]. In a trial of pembrolizumab in patients who were ipilimumab-naïve or who had previously received ipilimumab (on average 33–34 weeks prior; range, 4–248 weeks), the rates of grade 3–4 treatment-related AEs and the rates of patients discontinuing due to AEs were similar between ipilimumab-naïve and ipilimumab-treated patients: 14% *versus* 11% and 4% *versus* 5%, respectively [40, 48].

The trial investigating concurrent nivolumab plus ipilimumab also evaluated nivolumab following progressive disease and lack of grade 3–4 AEs in patients who had received ipilimumab in the previous 4–12 weeks [50]. In this cohort, 18% of patients experienced a grade 3–4 treatment-related AE and 9% of patients discontinued due to a treatment-related AE. As patients who had previously experienced high grade AEs with ipilimumab were excluded, the study did not assess the safety of sequential therapy in this patient population. While initial evidence suggests that PD-1 pathway checkpoint inhibitors can be used safely in patients with prior ipilimumab treatment, no data yet exists to describe the experience of patients treated with anti-CTLA-4 antibody following anti-PD-1 antibody. This is an area of ongoing investigation that will be of particular importance in future clinical practice.

Several fully-accrued trials are further investigating ipilimumab plus nivolumab in patients with advanced melanoma, and an expanded access program is now available [23]. A phase II study is evaluating the safety of sequential use of ipilimumab after nivolumab (four 3-week cycles of ipilimumab, followed by nivolumab every 2 weeks until progression or unacceptable toxicity) *versus* nivolumab after ipilimumab (six 2-week cycles of nivolumab followed by four 3-week cycles of ipilimumab followed by nivolumab every 2 weeks until progression or unacceptable toxicity) [NCT01783938]. A phase III study is evaluating nivolumab alone or with ipilimumab *versus* ipilimumab in patients with previously untreated advanced melanoma; the primary endpoint is OS [NCT01844505]. Another phase II study has a similar design, without the nivolumab alone group, and has ORR as the primary read-out [NCT01927419]. Other combinations of a CTLA-4-targeted agent plus a PD-1 pathway inhibitor are also being evaluated in phase I trials, including ipilimumab plus pembrolizumab or MPDL3280A, and tremelimumab plus MEDI4736 [23].

#### Strategies for managing AEs with checkpoint inhibitors

Detailed treatment algorithms and recommendations are available for the approved agent ipilimumab and nivolumab (Table 2 [12, 17, 21, 38, 40–46]). Patients should be examined for potential irAEs at each visit, and prompt work-up of suspected AEs should be performed to minimize the risk of worsening. Since asymptomatic grade 3–4 elevations in aspartate aminotransferase (AST) and/or alanine aminotransferase (ALT) have been noted in several studies of checkpoint inhibitors, clinical trials required that certain laboratory tests be performed at regular intervals, including those evaluating liver, renal, and thyroid functions [21, 38, 43, 45].

A particular AE to note with checkpoint inhibitors is diarrhea, which is due an inflammatory immune response, not off-target drug effects, as with chemotherapy or targeted agents [45]. Therefore, diarrhea induced by checkpoint blockade is treated with corticosteroids or other immunosuppressant(s) (Table 2 [12, 17, 21, 38, 40–46]). Similarly, most irAEs can be effectively managed with corticosteroid treatment; however, a prolonged taper is often required for complete resolution. General treatment strategies for irAEs are as follows:

- Grade 1–2 AEs are treated symptomatically, with increased frequency of monitoring.
- Grade 1–2 AEs that remain persistent or become more symptomatic should be managed similarly to grade 3–4 AEs.
- Grade 3–4 AEs should be treated with corticosteroids and tapered over 4 or more weeks [38, 42, 45, 46].

Endocrine disorders with checkpoint inhibitors have been managed with hormone replacement, which may or may not be permanent. Prolonged exposure to corticosteroid therapy, possibly to manage irAEs, may also lead to adrenal insufficiency and hypogonadism. Each of these supportive measures should be taken into consideration during the assessment of endocrinopathies [45]. Long-term exposure to corticosteroids can lead to infection, with opportunistic infections and gastrointestinal irritation [59]. Therefore, prophylactic antibiosis and gastric acid suppression may be indicated in patients requiring extended steroid tapers. The use of corticosteroids to manage irAEs does not appear to negatively impact the efficacy of checkpoint inhibitors, as tumor response duration appears to be unaffected in patients requiring this intervention [45]. In many patients, checkpoint therapy can be restarted after successful resolution of the AE [42, 48, 54]. However, patients who experience severe AEs should permanently discontinue treatment [38, 42, 45, 49].

### Patient selection

The identification of a selection marker for treatment, such as a *BRAF* mutation in melanoma, offers the ability to prospectively identify patients more likely to benefit from certain therapies. While ORRs with targeted therapies are high, not all patients are eligible. Initial evidence suggests that checkpoint inhibition may be more broadly applicable than targeted therapy. In trials of patients with melanoma being treated with checkpoint inhibitors, responses have been observed in patients with and without *BRAF* mutations, brain metastases, or prior treatment [12, 20, 40, 45, 57, 60]. As a potential biomarker of response, a rise in absolute lymphocyte count at 3, 7 or 12 weeks of ipilimumab treatment has been correlated with improved survival [61–64]. Studies have also shown a correlation between an increased eosinophil count—either at baseline or a rise between the second and third ipilimumab infusions—and improved survival [61, 65]. Also, an exploratory study found that pembrolizumab-treated patients with smaller baseline tumor size (≤ 90 mm) had higher responses and improved OS at 1 year as compared with patients with larger baseline tumors. However, patients with larger tumors also derived benefit from pembrolizumab [66]. Predictors of toxicity are also being evaluated with checkpoint inhibitors. For example, IL-17 levels at 7 weeks of treatment with ipilimumab predicted colitis [67]. Additionally, patients with a history of autoimmune disease may be more at risk for development of immunologic AEs with checkpoint inhibitors and were excluded from clinical trials [43, 50, 54].

Many trials are now investigating whether PD-L1 expression by tumors can be used as a predictive biomarker of response to PD-1 pathway inhibitors. Initial results suggest that response rates with PD-1 inhibitor monotherapy may be higher in patients with PD-L1-positive melanoma *versus* PD-L1 negative melanoma [20, 46, 57, 68]. However, in nearly all studies, responses were also seen in patients with negative or low PD-L1 tumor expression. It is likely that PD-L1 expression will be associated with an improved response rate with single agent PD-1 inhibition. Nevertheless, tumor PD-L1 expression will not discriminate against patients unable to benefit. In patients receiving concurrent CTLA-4 and PD-1 pathway inhibitors, high proportions of patients responded, regardless of baseline PD-L1 status (ORR: 57% for PD-L1 positive; 35% for PD-L1 negative) [57]. Therefore, tumor PD-L1 expression may not be useful as a prognostic biomarker for patients receiving combination regimens. Further evaluation of potential biomarkers is needed, with one option being evaluation of the inflamed *versus* non-inflamed tumor phenotype [69, 70].

## CONCLUSION

Checkpoint inhibitors, such as ipilimumab, anti-PD-1, and anti-PD-L1 antibodies, have emerged as new treatment modalities for patients with melanoma, and likely various other cancers. For a subset of patients treated with checkpoint inhibitors, durable clinical responses lasting many years may be possible. Immunotherapy combinations have shown increased efficacy and toxicity compared with monotherapy; however, to date, most toxicities have been manageable. Clinical trials are underway to examine various combinations and sequencing of ipilimumab and PD-1 pathway blockers, and it remains to be seen if sequential administration of immuno-oncology agents will be as efficacious or exhibit an improved (or worsened) toxicity profile. As such, treatment of patients with combinations or sequential approaches will require the close attention of clinicians for the development of immune-related toxicities. The understanding that immunologic AEs are caused by uncontrolled off-target immune responses, and therefore frequently require active treatment with steroids, is critical for clinicians to effectively manage patients receiving these therapies.
